# High-Pressure Near-Infrared Luminescence Studies of Fe^3+^-Activated LiGaO_2_

**DOI:** 10.1021/acs.inorgchem.3c01627

**Published:** 2023-07-27

**Authors:** Ajeesh Kumar Somakumar, Lev-Ivan Bulyk, Volodymyr Tsiumra, Justyna Barzowska, Puxian Xiong, Anastasiia Lysak, Yaroslav Zhydachevskyy, Andrzej Suchocki

**Affiliations:** † Institute of Physics, Polish Academy of Sciences, Al. Lotników 32/46, 02-668 Warsaw, Poland; ‡ Institute of Experimental Physics, Faculty of Mathematics, Physics and Informatics, University of Gdańsk, Wita Stwosza 57, 80-308 Gdańsk, Poland; § The State Key Laboratory of Luminescent Materials and Devices, South China University of Technology, Wushan Road 381, Guangzhou 510641, China

## Abstract

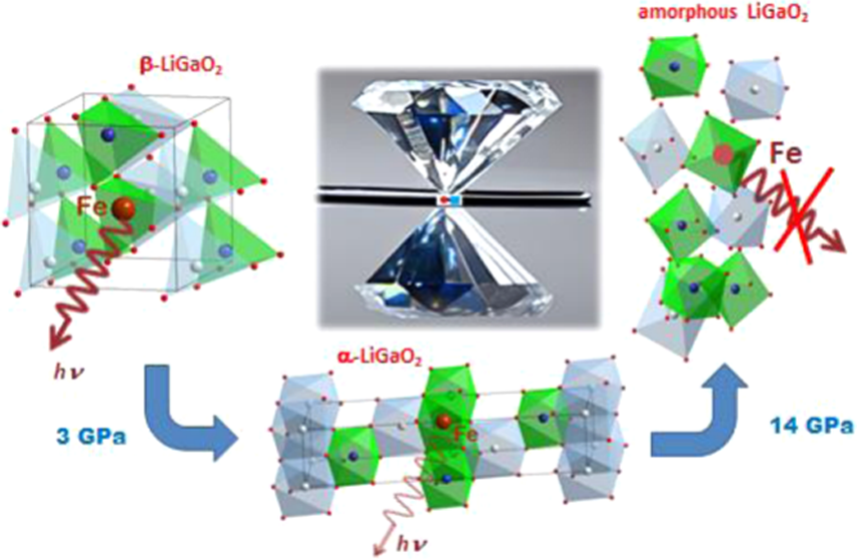

A 0.25% iron (Fe^3+^)-doped LiGaO_2_ phosphor was synthesized by a high-temperature solid-state reaction method. The phosphor was characterized utilizing X-ray diffraction (XRD), scanning electron microscopy (SEM), high-pressure photoluminescence, and photoluminescence decay measurement techniques using diamond anvil cells (DACs). The powder X-ray analysis shows that the phosphor is a β polymorph of LiGaO_2_ with an orthorhombic crystallographic structure at room temperature. The SEM result also confirms the presence of well-dispersed micro-rod-like structures throughout the sample. The photoluminescence studies in the near-infrared (NIR) range were performed at ambient, low-temperature, and high-pressure conditions. The synthesized phosphor exhibits a photoluminescence band around 746 nm related to the ^4^T_1_ → ^6^A_1_ transition with a 28% quantum efficiency at ambient conditions, which shifts toward longer wavelengths with the increase of pressure. The excitation spectra of Fe^3+^ are very well fitted with the Tanabe–Sugano crystal-field theory. The phosphor luminescence decays with a millisecond lifetime. The high-pressure application transforms the β polymorph of LiGaO_2_ into a trigonal α structure at the pressure of about 3 GPa. Further increase of pressure quenches the Fe^3+^ luminescence due to the amorphization process of the material. The prepared phosphor exhibits also mechanoluminescence properties in the NIR spectral region.

## Abstract 2

A Diamond Anvil cell was used for high-pressure studies of LiGaO_2_:Fe powder, emitting light in the near-infrared region with a 28% quantum efficiency at ambient conditions. The powder is composed of nano-crystallites with average size of about 32 nm. The high-pressure application transforms the βpolymorph of the LiGaO_2_ into a trigonal α structure at the pressure of about 3 GPa. Further increase of pressure quenches Fe^3+^ luminescence due to the amorphization process of the material.

## 1 Introduction

Several inorganic materials doped with iron (Fe^3+^) have broad absorption and emission spectra in the visible and infrared regions. One such compound is lithium gallate (LiGaO_2_), which displays a remarkably strong near-infrared (NIR) luminescence when doped with iron (Fe^3+^). LiGaO_2_ is a ternary semiconducting metal oxide compound with a direct bandgap.^1−4^ It crystallizes in at least four polymorphs α, β, γ, and δ.^5^ The β-LiGaO_2_ polymorph is particularly intriguing due to its stability under ambient pressure and temperature conditions. It shares a similar crystal structure to other well-known binary semiconductors such as GaN, ZnO, and CdSe, featuring a wurtzite-like structure where Li and Ga ions occupy the cation sublattices.^2,6^ To accommodate multiple Li and Ga atoms in the tetrahedral cages, the structure undergoes slight distortion from its original form, adopting a distorted wurtzite structure with the *Pna*2_1_ space group.^7^ Owing to its structural characteristics and larger bandgap value, LiGaO_2_ possesses several distinctive optical and mechanical properties when compared to materials with a classical wurtzite structure.

Transition-metal-doped LiGaO_2_ is commonly used as an efficient light-converting material. It shows a strong persistent luminescence under ambient conditions along with thermoluminescent and pyroelectric luminescent properties. Consequently, it is highly regarded as a promising phosphor material for fluorescent lamps in plant growth applications.^8−10^ Apart from its optical properties, solid β-LiGaO_2_ also demonstrates intriguing mechanical characteristics such as elasticity and piezoelectricity.^11^ β-LiGaO_2_ has also found applications as a ceramic tritium breeder material in experimental fusion reactors.^12,13^ It serves as a lattice-matched substrate for the growth of GaN, InN,^14^ and ZnO^15^ and as a solid gallium precursor source material for bulk GaN crystal growth.^16,17^ Recent studies have indicated the applicability of these ceramic phosphors in various biological fields due to their biocompatible nature and promising NIR luminescence properties when doped with transition-metal elements.^1,18^ Iron (Fe^3+^) is an efficient transition-metal dopant for achieving the NIR luminescence and persistent luminescence in LiGaO_2_.^19−21^

The bandgap of the material plays a crucial role in controlling its luminescence features for different applications. To effectively engineer the bandgap, it is important to understand the potential phase transitions that can take place within the material.^5^ Extensive research has confirmed that β-LiGaO_2_ exhibits a stable orthorhombic structural phase with a space group of *Pna*2_1_ under normal pressure. Employing a diamond anvil cell (DAC) for high-pressure luminescence studies offers a valuable approach to investigate the formation of new phases in the material. Additionally, the first-principles studies conducted on β-LiGaO_2_ under various high-hydrostatic-pressure conditions have indicated the occurrence of multiple phase transitions in this material.^22^ This paper presents the findings of our investigation, focusing on the different phase transitions observed in the β-LiGaO_2_ material doped with iron (Fe^3+^) at high hydrostatic pressure. We analyze its near-infrared luminescent properties under different physical conditions to gain insights into these transitions.

Furthermore, we have confirmed that LiGaO_2_:Fe possesses mechanoluminescent (ML) properties. To the best of our knowledge, no previous reports have documented ML in LiGaO_2_:Fe until now. The ML spectral range of LiGaO_2_:Fe fully overlaps with its photoluminescence spectrum, making it a very interesting and promising material for potential applications in bioimaging and phototherapy.

## 2 Experimental Section

### 2.1 Materials and Synthesis

The LiGaO_2_ is usually synthesized by the mixing of two metal oxides in stoichiometric ratios. The general reaction formula for LiGaO_2_ preparation is the following^23^
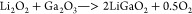
where the gallium (Ga^3+^) ions provided by the Ga_2_O_3_ having the coordination number (CN) = 4, forms the basic wurtzite structure. The LiGaO_2_:Fe^3+^ 0.25% sample used for the high-pressure studies in the present work is prepared by the method of a high-temperature solid-state reaction of the following chemical compounds reported in the literature.^1^ In this experiment, LiCO_3_, Ga_2_O_3_, and Fe_2_O_3_ are used as the main precursor materials, in which Fe_2_O_3_ will act as the Fe^3+^ ion donor to the phosphor. The abovementioned chemicals of high purity were mixed in an agate mortar and followed the same high-temperature synthesis method and conditions described in the literature.^1^ Initially, the prepared sample was preheated at 900 °C for 2 h using an alumina crucible and later calcined for 4 h further at 1000 to 1300 °C. Then, the prepared sample was kept at room temperature to cool down for further characterization. The results of characterization measurements of the sample studied here are presented in ref (1).

### 2.2 Experimental Techniques

The powder X-ray diffraction (XRD) measurements were performed with a BRUKER D2 PHASER using Cu Kα radiation operating at 30 kV and 10 mA. XRD patterns were collected with a 2θ value between 10 and 80° with a scan step of 0.02° and a counting time of 0.4 s per step, and phase analysis was performed using DIFFRAC.EVA V4.1 software from BRUKER. A Hitachi SU-70 scanning electron microscope (SEM) was used for analyzing the surface morphology of the synthesized sample. The room-temperature and low-temperature photoluminescence spectra were measured using a Horiba Fluorolog-3 modular spectrometer with a 450 W Xenon lamp excitation source and the sample compartment coupled with an external cryostat with a temperature controller. The quantum yield of the sample was measured with a Horiba “Quanta-φ” integrating sphere attachment on the same setup using a xenon lamp for excitation. The high-hydrostatic-pressure measurements were done using a diamond anvil cell (DAC) from easyLab Technologies Ltd. with diamonds of 0.45 mm culet size. The sample-holding gasket was prepared with an Inconel-718 metal alloy. A mixture of methanol and ethanol prepared in a 5:1 ratio was used as a pressure-transmitting medium. A small ruby (Al_2_O_3_:Cr^3+^) sphere was used as the standard pressure gauge. The R1-luminescence line from the ruby was used for the pressure calibration. The high-pressure measurements were performed on a Horiba Jobin-Yvon FHR 1000 monochromator with a liquid nitrogen-cooled CCD detector, and an Oxford Optistat CF cryostat is used for mounting the DAC for low-temperature measurement. A Coherent Innova 400 Ar-ion, 275.4 nm ultraviolet (UV) laser excitation was used to excite the LiGaO_2_:Fe^3+^ sample. The high-pressure luminescence decay measurements were performed using a pulsed Nd:YAG optical parametric oscillator (OPO) laser excitation source from EKSPLA and an Acton Spectra Pro SP-2500 monochromator from Princeton Instruments with an SR430 Multi-channel scaler (Stanford Research Systems) with a photon-counting system.

A custom-built setup controlled by dedicated software in a LabView environment was used to measure the friction-induced mechanoluminescence (ML). The sample was fixed to a PMMA plate with specially selected adhesive tape. ML was induced by the glass rod mounted on a linear rail. The rod was pressed toward the sample with a preset force and made a preset number of movements at a preset speed. The ML signal was collected using a Shamrock 500i spectrometer with a TEC iDus 420 camera (Andor Technology).

## 3 Results

### 3.1 XRD and SEM Results

**Figure 1:**
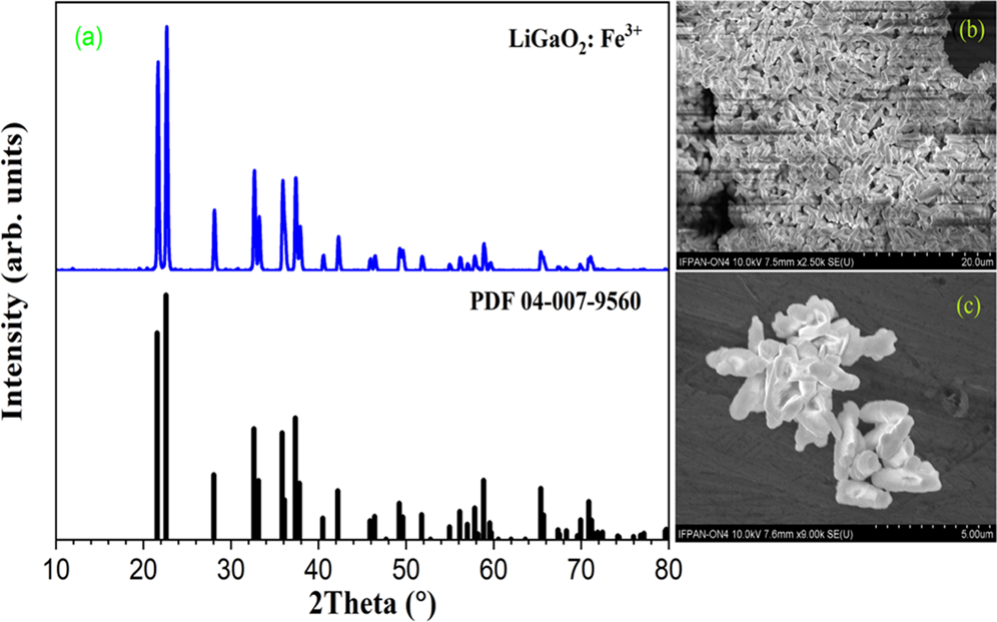
(a) X-ray diffraction pattern of the studied LiGaO_2_:Fe^3+^ sample at ambient conditions (upper) and the standard LiGaO_2_ PDF card number 04–007–9560 (lower), (b) SEM image of the β-LiGaO_2_ sample, and (c) enhanced SEM image of the part of the sample.

The X-ray powder diffraction (XRPD), see Figure 1a, analysis shows that well-crystallized formations are present in the synthesized powder at ambient conditions. XRD peak data analysis also confirms that the powder has an orthorhombic phase at ambient pressure conditions with cell dimensions *a* = 5.402 Å, *b* = 6.372 Å, *c* = 5.007 Å, *a*/*b* = 0.8477, and *c*/*b* = 0.78578. The structure has a space group of *Pna*2_1_. The XRPD data are well in line with the standard XRPD data reported for β-LiGaO_2_ by Marezio.^24^ There is a small peak of 2θ value around 30.8°, which is more closely related to the low traces of LiGa_5_O_8_ present in the sample. The detailed analysis shows that the measured sample contains 0.8% LiGa_5_O_8_ phase (based on the PDF 04–002–8232 card number). The ionic radius of the Ga^3+^ ion in tetrahedral coordination is 0.047 nm and the dopant Fe^3+^ used in this study has an ionic radius of 0.049 nm,^25^ so the incorporation of Fe^3+^ in the place of Ga^3+^ does not cause much change in the basic structure of the sample due to their relatively close ionic radii. Here, the XRD data show that the Fe^3+^ is perfectly incorporated into the sites of Ga^3+^ without causing any change in the basic structure of the β-LiGaO_2_ sample. The average crystalline sizes of the crystallites in the sample were calculated from the full width at half-maximum (FWHM) and the Debye–Scherrer equation
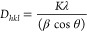
1where *D_hkl_* is the size of the crystallite, β is the full width at half-maximum (FWHM), and θ is the Bragg angle. Obtained *D_hkl_* is equal to about 32.0 nm, which points toward the nanosize distribution of crystallites in the powder sample.

The surface morphology analysis of β-LiGaO_2_:Fe^3+^ using a scanning electron microscope (SEM) shows that the synthesized sample has some unique morphological features. Figure 1b shows that the β-LiGaO_2_:Fe^3+^ crystallites of nanosize further fused forming large micro-rod-like structures of almost similar sizes. The image also confirms that these structures are well dispersed all over the sample. The enhanced image in Figure 1c shows how a bundle of a few microrods looks together. These microrods have an average dimension of 0.5 to 1 μm.

### 3.2 Ambient Pressure Spectroscopy

**Figure 2:**
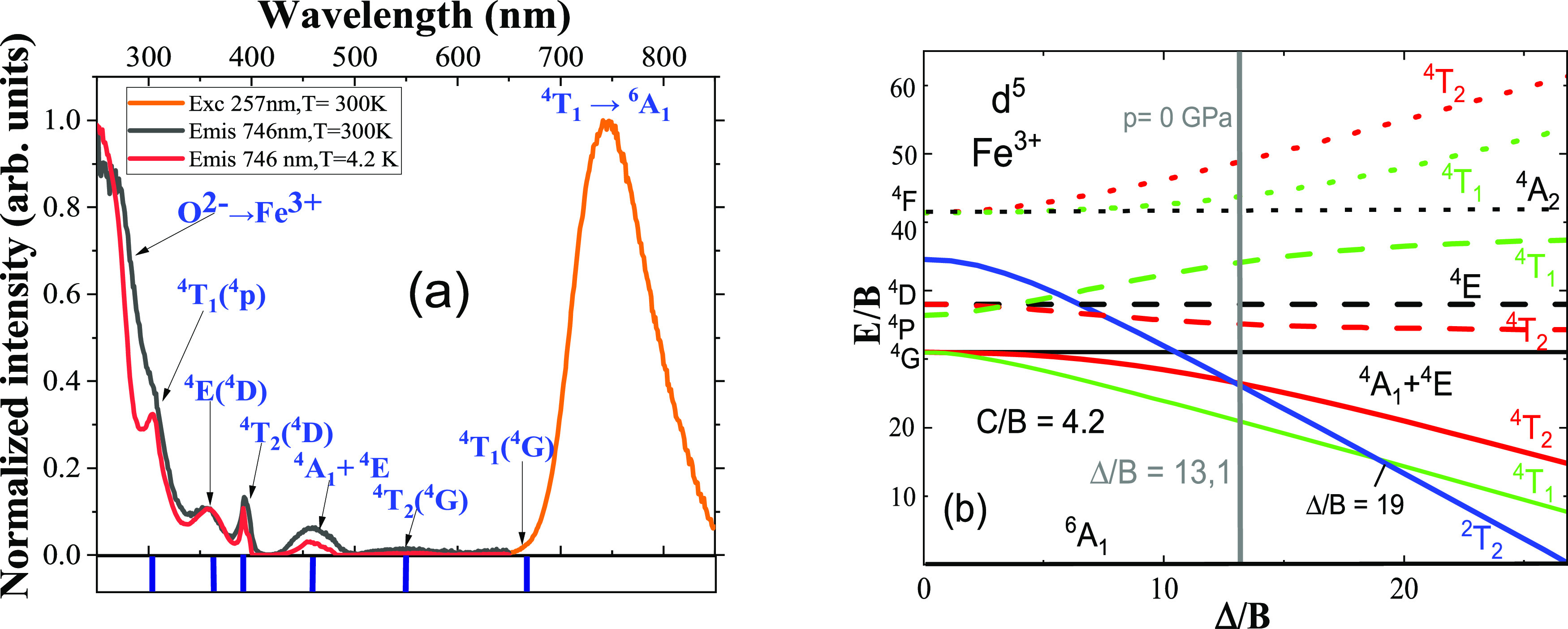
(a) Photoluminescence excitation spectra of LiGaO_2_:Fe^3+^ at ambient (black line) and low-temperature (red line) conditions and photoluminescence emission spectra (yellow line) at ambient temperature, (b) partial Tanabe–Sugano diagram for the d^5^ electronic configuration with Racah *B*, *C*, and crystal-field strength *Dq* parameters from the fit of crystal-field theory to the PLE spectrum (for the d^5^ configuration, the Tanabe–Sugano diagram is the same for octahedral and tetrahedral coordination).

The crystallographic structure study of β-LiGaO_2_ in the literature shows that its cations are tetrahedrally coordinated. The material is reported to have bandgap values of around 5.6 eV at room temperature and 6.25 eV at low temperature.^26^ These values are larger than those for many wurtzite materials. In this context, the luminescence study of β-LiGaO_2_ using an efficient transition-metal ion dopant like Fe^3+^ in ambient and low-temperature conditions can provide valuable information about the electronic structure of the dopant. The energy of the Fe^3+^ luminescence in the β-LiGaO_2_ significantly depends on this crystal-field strength (CFS) experienced by the Fe^3+^ ion from its O^2–^ ligands surrounding.^27^ The crystal-field theory and Tanabe–Sugano (T–S) diagrams explain the energy structure and crystal-field strength of transition-metal ions in the sample. The d^5^ configuration Tanabe–Sugano (T–S) diagram, constructed with fitted Racah *B, C*, and crystal-field strength parameters *Dq* is presented in Figure 2b.

Figure 2a shows the excitation and emission spectra of LiGaO_2_:Fe^3+^ recorded at ambient pressure and temperature conditions together with the excitation spectrum measured at low temperatures. The photoluminescence excitation (PLE) spectrum is dominated by the very strong and broad charge-transfer (CT) band in the UV region below 350 nm related to the O^2–^ → Fe^3+^ transition. The remaining weaker peaks are related to internal transitions from the ground ^6^A_1g_ state to the quartet excited states of the Fe^3+^ion. The first six quartet excited states can be distinguished in the spectra. Their designation is shown in Figure 2a, and the peak energies are listed in Table 1.

**Table 1:** Position of Lowest Quartet Excitation States of the Fe^3+^ Ion in LiGaO_2_ Obtained from Experiment and the Fit of the Crystal-Field Theory

terminating state	wavelength and energy of the PLE peak	theoretical energy from the fit of T–S matrices
^4^T_1g_ (^4^G)	668 nm (14,970 cm^–1^)	14,951 cm^–1^ (669 nm)
^4^T_2g_ (^4^G)	550 nm (18,180 cm^–1^)	18,895 cm^–1^ (529 nm)
^4^A_1g_ + ^4^E_g_ (^4^G)	460 nm (21,740 cm^–1^)	22,099 cm^–1^ (453 nm)
^4^T_2g_ (^4^D)	393 nm (25,450 cm^–1^)	25,040 cm^–1^ (399 nm)
^4^E_g_ (^4^D)	360 nm (27,780 cm^–1^)	27,090 cm^–1^ (369 nm)
^4^T_1g_ (^4^P)	304 nm (32,900 cm^–1^)	31,443.57 cm^–1^ (318 nm)
CT (O^2–^ → Fe^3+^)	∼250 nm (40,000 cm^–1^)	

According to T–S theory, the energies of ^4^A_1_ and ^4^E bands are given in terms of Racah parameters *B* and *C* by the following formulas
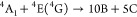
2
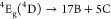
3The energies of the ^4^T states are given by the solution of the 3rd order nonlinear equations and expressed in terms of Racah and crystal-field strength Dq parameters. The most probable values for Δ*B* and *C* obtained from the fit of the crystal-field theory are the following: Δ = 9611 cm^–1^, *B* = 713 cm^–1^, *C* = 2995 cm^–1^, Δ/*B* = 13.1, and *C*/*B* = 4.2. The obtained energies of the CF levels are listed in Table 1 as theoretical results and presented at the bottom of Figure 2a. The agreement between the theoretical fit and the experimental positions of the PLE bands is very good. Experimentally observed values of the Racah and Dq parameters are similar to the obtained values for the other materials containing Fe^3+^ (see, for example, ref (28)), although sometimes there are difficulties in proper excitation (or absorption) band designation. Theoretical values of the free-ion Racah parameters are much higher, as, for example, found in ref (29), *B*_0_ = 1130 cm^–1^ and *C*_0_ = 4111, and those calculated in ref (30) are equal to 1296 and 4826 cm^–1^, respectively. Experimental values of the free-ion Racah *B* and *C* parameters are different than calculated theoretically^31^ and equal to *B*_0_ =814 cm^–1^ and *C*_0_ = 4932 cm^–1^. The reduction of the Racah parameters in the host environment is a result of the covalence effect, which is expressed by the so-called nephelauxetic parameter β_1_^32^
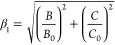
4The calculated β_1_ parameter using eq 4 is equal to 1.07.

In contrast to the observed transitions to the different quartet states, the experimental data do not show evidence of the transition between the ^6^A_1_ ground state to the ^2^T_2_ excited state in the excitation spectra. The absence of this peak in the excitation spectra is due to the high spin-forbidden nature of the ^6^A_1_ → ^2^T_2_ transition. and probably they are hidden under the transitions to the quartet states. Excitation in any of the PLE bands induces the same emission with a peak maximum of around 746 nm related to the transition from the ^4^T_1_ excited state to the ^6^A_1_ ground state of the d^5^ configuration. The photoluminescence quantum yield measurement of β-LiGaO_2_:Fe^3+^ phosphor was recorded at ambient conditions showing the QE value of around 28% when the phosphor was excited through the CT band. Only 8% QE was recorded when the luminescence is excited through the ^6^A_1_ → ^4^T_2_(^4^D) transition at 393 nm.

**Figure 3:**
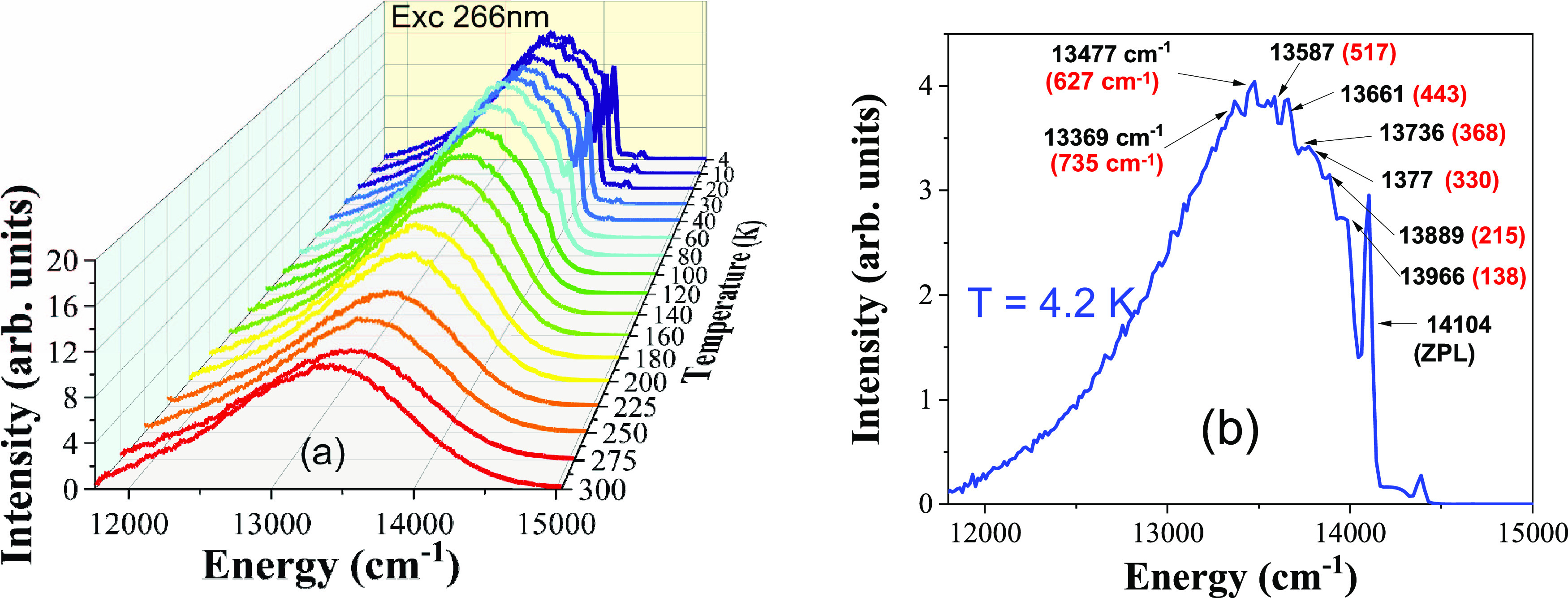
(a) Temperature dependence of photoluminescence emission spectra of the LiGaO_2_:Fe^3+^ sample at ambient pressure and (b) photoluminescence emission spectra of LiGaO_2_:Fe^3+^ phosphor at 4.2 K; the energy difference between zero-phonon line and phonon replicas are marked in red. All energies are given in cm^–1^.

Figure 3a shows the temperature-dependent emission spectra of LiGaO_2_:Fe^3+^ phosphor. The emission spectra were recorded at a temperature ranging from 4.2 to 300 K. The sample was excited into the same broad 266 nm CT band. In that figure, we can also observe that the overall intensity of the main band decreases with an increase in temperature. A sharp zero-phonon line (ZPL) around 709 nm is visible between 4.2 and 100 K. The intensity of this 709 nm sharp ZPL decreases with the increase in temperature. From 100 K to around 300 K, the shapes of emission spectra are very similar, except for the change in intensity and a small change in position.

The sharp ZPL related to the Fe^3+^ ions is observed at a wavelength of around 709 nm. Its presence at low temperatures suggests that the Fe^3+^ dopant ion interacts relatively weaker with the host lattice compared with other compounds, for example, isoelectronic Mn^2+^ ions in several materials. A phonon sideband structure is observed on the top of the main luminescence band around 746 nm at low temperatures. In addition to that, a small unknown peak structure is visible around 695 nm at low temperatures, which may be related to the unintentional chromium impurity (Cr^3+^) present in the sample^33^ or to another small concentration Fe^3+^ center,^34^ possibly related to another LiGa_5_O_8_ perovskite phase, observed in the XRD experiment.

Figure 3b shows the ^4^T_1_ → ^6^A_1_ transition-related low-temperature emission spectra of Fe^3+^ in the LiGaO_2_ sample at 4.2 K. The energies of the ZPL (14,104 cm^–1^) and the phonon replicas are marked on the main luminescence band and the difference between the energies of the ZPL and each phonon replica are marked in the spectra with red color (cm^–1^) in the parentheses. Table 2 shows the comparison of energy difference obtained from the present low-temperature photoluminescence experiment (column-1) and the reference data from a previous experimental Raman study of β-LiGaO_2_ under ambient pressure (column-2).

**Table 2:** Comparison of the Peak Energy Difference Obtained from the Low-Temperature Photoluminescence and the Raman Phonon Frequencies Obtained from the Experimental Raman Study of LiGaO_2_

difference between the energy of the zero-phonon line (ZPL) and phonon replicas (cm^–1^)	experimentally observed from the Raman study of LiGaO_2_^35^ (cm^–1^)	Raman mode designation
138	128.7	A_1_^(1)^
215.6	204.2	B_1_^(1)^
330.3	289.0	A_2_^(1)^
368.1	*	*
443.2	444.3	A_1_^(3)^
517.4	502.1	A_1_^(5)^
627.4	643.9	A_1_^(6)^
735.4	*	*

Table 2 shows that the energy values obtained from both photoluminescence and Raman measurement are close to each other. The peak energy values 368.1 and 735.4 cm^–1^ from the photoluminescence have no analogues found in the Raman data of β-LiGaO_2_. These two phonon replicas probably originate from the combination of some other lower-energy phonons.

**Figure 4:**
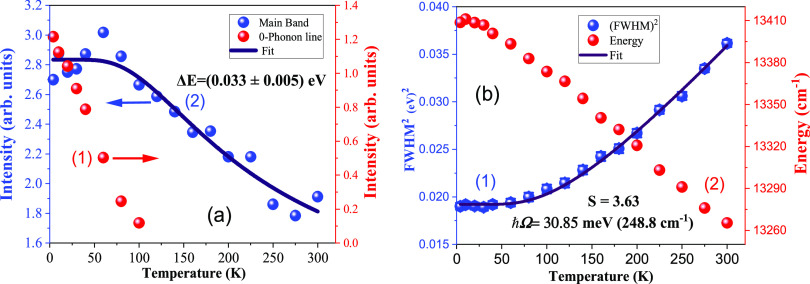
(a) Integrated emission intensity versus temperature graph of LiGaO_2_:Fe^3+^ powder: (1) ZPL, where red points represent the experimental data and (2) main band (blue points represent the experimental data points and the black line is the fit of eq 5); (b) temperature dependencies of (1) full width at half-maximum of the main luminescent band (blue points with a black fitting line) and (2) PL peak energy (red points).

Figure 4a shows the integrated emission intensity as a function of the temperature of the main emission band and the zero-phonon line of the LiGaO_2_:Fe^3+^ powder. The graph was plotted by integrating the emission spectra of the sample shown in Figure 3a. It shows that the intensity nearly doubles at low temperatures. The PL total emission intensity and temperature (K) relation is generally described by the Arrhenius equation
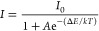
5where *I*_0_ represents the intensity at low temperature, *k* is the Boltzmann constant, and Δ*E* is the activation energy. Its value obtained from fitting is equal to Δ*E* = (0.033 ± 0.005) eV. This result shows that for temperature nonradiative luminescence quenching of Fe^3+^ ions, a quenching level located about 33 meV above the luminescent ^4^T_1g_ state is most probably responsible.

Figure 4b shows the change in energy and full width at half-maximum (FWHM) of the main luminescence band with respect to temperature. The peak position is slightly red-shifted to longer wavelengths (around 10 nm) and the FWHM of the main luminescence band increases. Figure 4b shows the fitted graph of FWHM with respect to temperature. The fit was obtained using the following equation^36,37^
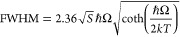
6where ℏΩ is the effective phonon energy, k is the Boltzmann constant, and S is the Huang–Rhys factor, which is a measure of linear electron–phonon coupling strength.^38^ Calculations of the fitted data show that the effective phonon energy ℏΩ = 30.85 meV (or 249 cm^–1^), which agrees with experimental Raman data mentioned in Table 2. The Huang–Rhys factor is equal to *S* = 3.36, which indicates the relatively weak electron–phonon coupling for the LiGaO_2_:Fe^3+^. The experimental Stokes shifts observed for the absorption and emission spectra at low temperatures mainly depend on the value of the Huang–Rhys factor. The Stokes shift observed from the spectra is calculated from the following relation:
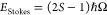
7The calculations using eq 7 show that the Stokes shift value is equal to *E*_Stokes_ = 1558 cm^–1^. The experimentally observed ^4^T_1g_ (^4^G) → ^6^A_1g_ broad emission band is around 746 nm (13405 cm^–1^). If we add the above-obtained Stokes shift value to the experimental ^4^T_1g_ (^4^G) → ^6^A_1g_ emission band energy, the expected absorption peak should be around 668 nm (14963 cm^–1^), which is in good agreement with the experimentally and theoretically calculated values of the ^6^A_1g_ → ^4^T_1g_ (^4^G) band according to Table 1.

### 3.3 High-Pressure Luminescence Studies

**Figure 5:**
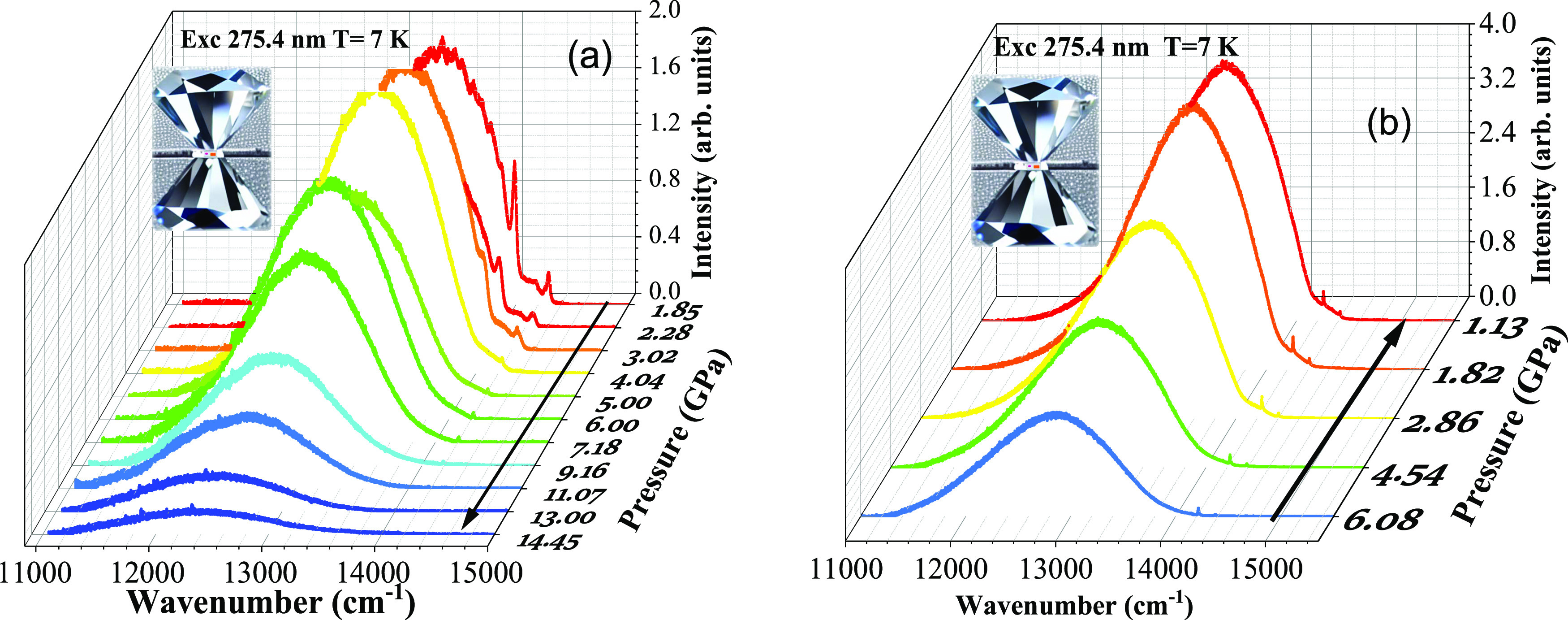
Pressure dependence on the emission spectra of LiGaO_2_:Fe^3+^powder at 7 K; (a) compression and (b) pressure release.

**Figure 6:**
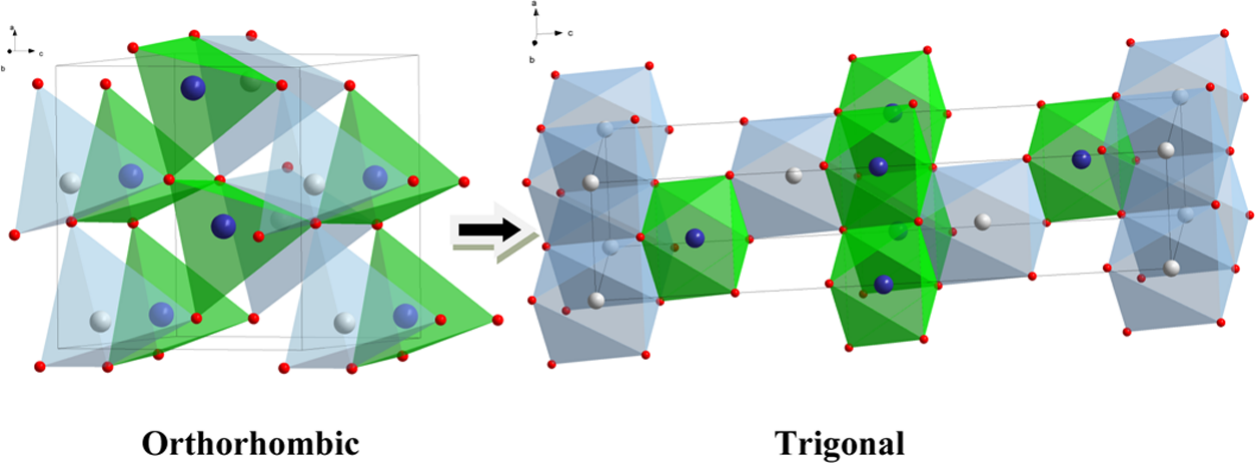
Elementary unit cells of orthorhombic β-LiGaO_2_ (below 3 GPa) and trigonal α-LiGaO_2_ (above 3 GPa) structures. Ga^3+^ ions are marked blue, Li^+^—gray, and oxygen O^2–^—red. It is seen that the tetrahedrally coordinated environment of Ga^3+^ ions transforms to octahedral with the increase of pressure.

Figure 5a shows the high-pressure luminescence spectra LiGaO_2_:Fe^3+^ sample at T = 7 K. At the initial pressure of about 1.85 GPa, the spectra look similar to the low-temperature spectra measured at ambient pressure. After increasing the pressure, the phonon line at 709 nm (14,104.4 cm^–1^) intensity decreases and it completely disappears around 4 GPa. That points toward a certain phase transformation occurring around 3 GPa. This high-pressure phase of LiGaO_2_ around 3 Gpa and at a temperature of around 850 °C was observed and reported earlier using an X-ray crystallographic study of LiGaO_2_ under high pressure. It was reported that a stable orthorhombic phase of LiGaO_2_ changed to the trigonal phase (space group *R*3̅*m*)^22,39^ at higher hydrostatic pressures between 1.4 and 3.7 GPa. The disappearance of the sharp zero-phonon line related to the Fe^3+^ ion in the phosphor also hints toward this phase transformation. Figure 6 shows the schematic illustration of LiGaO_2_ structures at ambient and higher than 3 GPa pressures. Figure 6 shows also the change in the coordination of the Ga^3+^ ion in the LiGaO_2_ related to pressure-induced phase transformation. Initially, at ambient pressure, the Ga^3+^ ion in LiGaO_2_ has a tetrahedrally coordinated environment, but after applying pressure of around 3 GPa, its tetrahedral coordination changes to octahedral due to the orthorhombic to trigonal phase transformation. Previous crystal structure studies of β-LiGaO_2_ at ambient pressure and temperature conditions show that in tetrahedral coordination, the Ga–O bond length is around 1.835 ± 0.004 Å. There is no such data for the high-pressure phase with tetrahedral coordination, at room temperature, but refs (24, 39, 40) report the value of 2.0 ± 0.01 Å for the bond length, when the sample was heated up to 850 °C.

**Figure 7:**
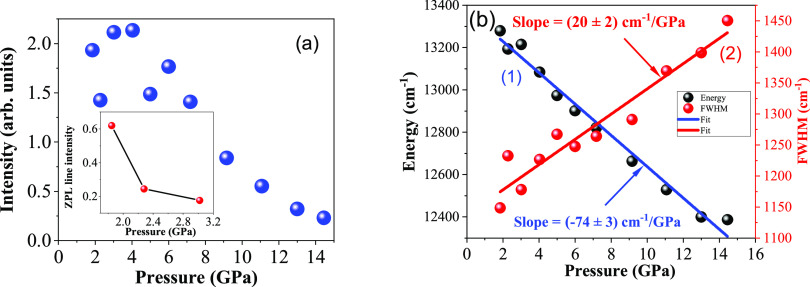
(a) Dependence of intensity of the main emission band and emission intensity of the zero-phonon line (inset) as a function of pressure for the LiGaO_2_:Fe^3+^ phosphor. (b) (1) PL peak energy versus pressure and (2) FWHM versus pressure graphs of LiGaO_2_:Fe^3+^ powder.

Further increase of pressure induces a strong decrease of the luminescence intensity. Figure 7 shows the luminescence intensity of the LiGaO_2_:Fe^3+^ as a function of pressure, and the inset graph in Figure 7a shows the quenching of zero-phonon line intensity around 3 GPa. The overall intensity decreases with the pressure increase which can be especially well observed at pressures above 6 GPa. Around 14.45 GPa, the sample luminescence was almost completely quenched. The reasons for the observed effect will be discussed in Section 4 of the paper. With the increase of pressure, the transition related to the main emission band between the lowest quartet ^4^T_1g_ and the ^6^A_1_ ground-state level moves toward a higher crystal field in the Tanabe–Sugano diagram (Figure 2b). The energy of this ^4^T_1g_ level gradually decreases with an increase in crystal-field strength.

Figure 7b shows the PL peak energy and FWHM of the main luminescence band as functions of pressure. The black points on Figure 7b show that the main peak red-shifts linearly to lower energies (longer wavelength) with the increase in pressure. The pressure coefficient of PL energy is equal to around −74 cm^–1^/GPa. Similar behavior was also observed in the Mn^2+^-doped pentaborate sample under high pressure.^41^ As well, due to the increase in pressure, the position of the low-temperature phonon line is slightly red-shifted, from its initial position of 709 to 714 nm. This is related to the pressure-induced increase covalency of the material. The FWHM of the luminescence increases with pressure, which is shown in Figure 7b. During the high-pressure measurement, we observed that the multiple phase transitions in β-LiGaO_2_:Fe^3+^ have an irreversible nature because the phosphor luminescence intensity does not recover its initial value after the release of pressure. To further confirm that the low-pressure phase is also irreversible, we repeated the experiment with a fresh sample mounted in the same DAC, then increased its pressure to 7.38 GPa, and then slowly released it. Figure 5b shows the pressure-releasing effect on the sample emission spectra. It is important to note that the sample main luminescence band ^4^T_1_→ ^6^A_1_ gets back to its initial position but did not get back its initial emission intensity after releasing the pressure completely. Moreover, it did not get back its initial 709 nm ZPL observed earlier at low-pressure and low-temperature conditions. That also confirms that the proposed low-pressure phase transition of Fe^3+^-doped LiGaO_2_ is an irreversible one. Figure 5b presents a lack of ZPL in the luminescence spectra after pressure released to 1.13 GPa.

**Figure 8:**
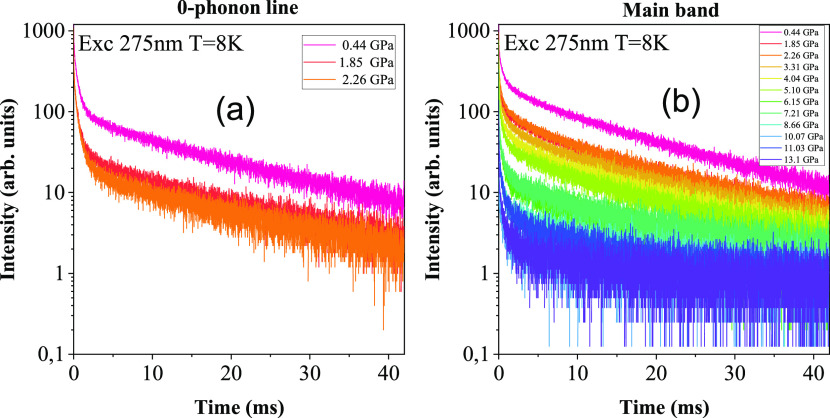
Luminescence decay profile of (a) zero-phonon line and (b) main band with respect to pressure.

**Figure 9:**
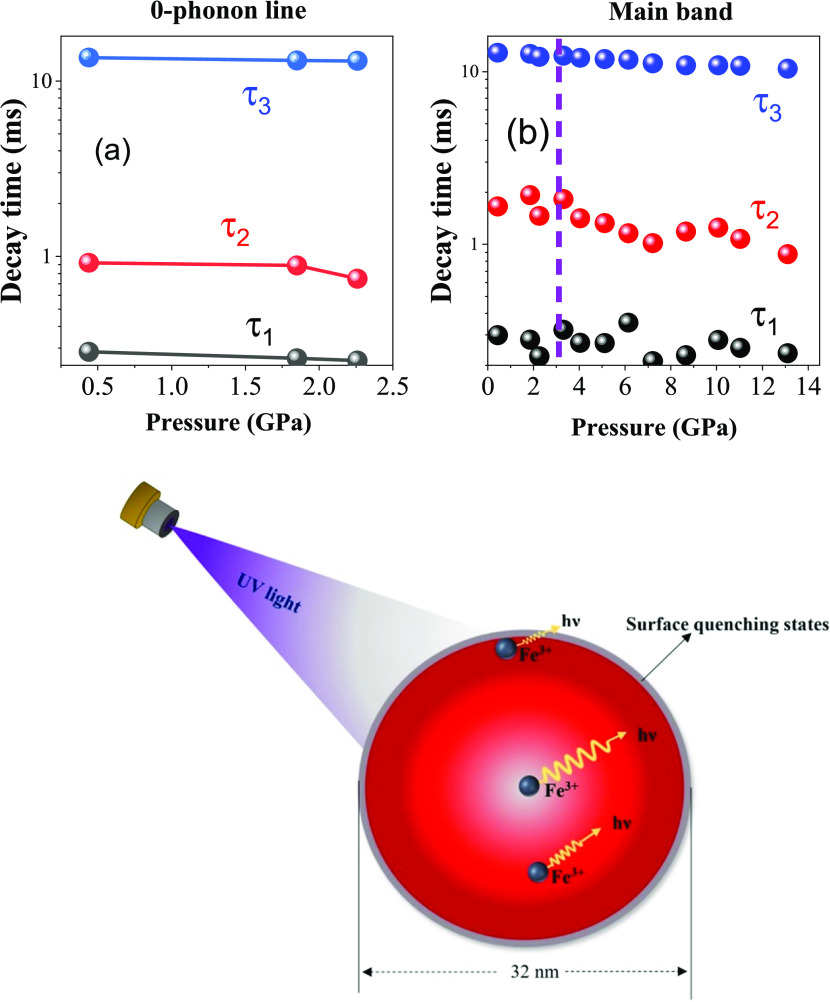
Luminescence decay time (for (a) zero-phonon line and (b) main band) as a function of the pressure of LiGaO_2_:Fe^3+^ powder. The vertical dotted line in panel (b) denotes the location of the observed phase transition. Bottom graph: Graph depicting the origin of three-exponential Fe^3+^ luminescence decays.

The results of luminescence decay measurements are presented in Figure 8. The decay measurements were taken from both the zero-phonon line (until it is observed) and the main luminescence band using a 275 nm laser excitation, and the time dependence of the luminescence intensity, *y*_0_, was fitted with the triple exponential equation
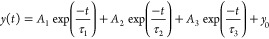
8where *A*_1_, *A*_2_, and *A*_3_ are the luminescence intensities of the particular decay component at time *t* = 0 and τ_1_, τ_2_, and τ_3_ are the decay times, respectively, and *y*_0_ is a constant component related to the electronic background. Initially, the decays were measured for both luminescence bands at 709 nm (zero-phonon line) and 745 nm (main band). Figure 9a shows, as in the high-pressure luminescence measurement (Figure 5a), the ZPL disappears around 3 GPa pressure at 8 K temperature. During the initial measurements at 0.44 GPa pressure, the fitted decay time values of the phonon band show τ_1_ = 0.28 ± 0.01 ms, τ_2_ = 0.92 ± 0.01 ms, and τ_3_ = 13.6 ± 0.2 ms, and the main band show τ_1_ = 0.29 ± 0.01 ms, τ_2_ = 1.66 ± 0.1 ms, and τ_3_ = 12.84 ± 0.2 ms. The presence of multiple luminescence lifetime values indicates the occupancy of Fe^3+^ ions in different lattice sites of the nanocrystallites. The longest component is probably related to the Fe^3+^ ions occupying lattice sites inside the nanocrystallites, and shorter components of the decay time are probably related to the Fe^3+^ ions close to the surface sites. The energy transfer between the Fe^3+^ ions and the luminescence quenching centers on the surface of the nanocrystal is the reason behind the shortening of decay time, which is depicted in Figure 9c. However, it seems that the distribution of the distances between the Fe^3+^ ions, which act here as the energy donors, and the luminescence quenching centers, presumably on the surface of the nanocrystallites, acting as the excitation energy acceptors, is not statistical. An attempt fitting of the Fe^3+^ decay kinetics with the Inokuti–Hirayama equations^42,43^ does not yield acceptable fits. Therefore, we fitted the decay kinetics with the three-exponential decay curves, which give much better fits.

The small size of nanocrystallites (in accordance with our XRD data) forming the LiGaO_2_:Fe powder is responsible for a relatively large contribution of the short components of the luminescence decay to the overall decay kinetics of this compound. Detection of the Fe^3+^ ions which undergo nonradiative quenching explains the relatively low quantum efficiency of LiGaO_2_:Fe^3+^, observed especially at room temperature. The long decay time of around 13 ms observed for undistorted Fe^3+^ ions is due to the spin-forbidden character of the ^4^T_1_ → ^6^A_1_ transition.^44^

The decay kinetics excited by the longer wavelengths in the internal absorption bands of Fe^3+^ ions (around 393 and 460 nm), associated with ^6^A_1_→(^4^T_2g_ (^4^D), ^4^E_g_ + ^4^A_1g_ (^4^G)) transitions, are similar to those excited in the charge-transfer band; however, the observed luminescence is much weaker, in accordance with the photoluminescence excitation spectrum. This means that the longest component of the decays is associated with the decays of Fe^3+^ ions, and it is not affected by the weak persistent luminescence observed in this material.^1^

Figure 9 shows the decay time versus pressure graphs of both the ZPL and the main band. The graphs show that the decay time slowly decreases with respect to the increase in pressure. The similarity in decay time (Figure 9a,b) values suggests that both luminescence peaks evolved from the same Fe^3+^ ion centers in the sample. This is well in line with the decay measurement values reported previously for the Fe^3+^-doped oxide materials in the literature, which is ranging from 1 to 40 ms.^1,45,46^ The decay times of the longest component decrease from about 13 ms at low pressure to about 10.3 ms at 14 GPa. The decay times of the shorter components also decrease slightly with the increase of pressure.

### 3.4 Mechanoluminescence

LiGaO_2_:Fe^3+^ (and also other lithium gallate oxides such as LiGa_5_O_8_:Cr)^47^ exhibits a well-discernible mechanoluminescence, which is most probably related to the existence of various traps, which can store charges excited by UV irradiation, especially in the charge-transfer spectral region. This effect is interesting since ML occurs in the near-infrared region, i.e., in the first biological window.

**Figure 10:**
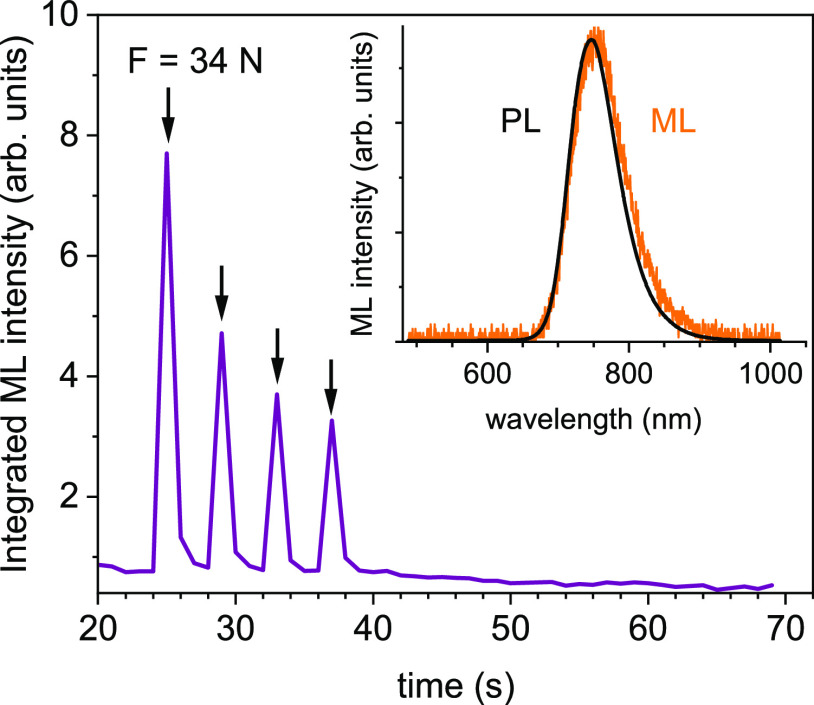
Integrated mechanoluminescence signal. Arrows indicate moments when the force of 34 N was applied to the sample. Inset—mechanoluminescence (ML) and photoluminescence (PL) spectra measured at room temperature.

Figure 10 shows the integrated mechanoluminescence signal as a function of the time elapsed since the end of sample irradiation. Before the ML experiment, the sample was irradiated for 5 min with 280 nm light from a diode and then kept in the dark for 25 s, after which the ML experiment was performed. To induce ML, the glass rod was pressed toward the sample plate with a force of 34 N and made four movements with a speed of 6 mm/s, every 4 s, each time drawing on the same part of the sample plate. As one can see in Figure 10, the sample LiGaO_2_:Fe^3+^ responded to the applied mechanical loading emitting ML, although the intensity of ML decreased with successive rod movements.

The inset shows a comparison of the ML spectrum with the PL spectrum measured at room temperature and assigned to the transition ^4^T_1g_ → ^6^A_1g_ in Fe^3+^ dopant ions. The spectral range and shape of the ML spectrum are very similar to the PL spectrum, although the ML spectrum appears to be slightly shifted toward longer wavelengths (lower energies). The similarity of the ML and PL spectra indicates that the Fe^3+^ ions are the only centers through which the energy stored in the trap states is radiatively deactivated as a result of applied mechanical loading. The shift of the ML spectrum compared to the PL spectrum, about 125 cm^–1^, is consistent with the T–S diagram and the results obtained in experiments performed in DAC. Even though in the ML experiment the pressure was not hydrostatic like in the DAC experiment, the randomly arranged crystals of the sample were subjected to an axial pressure of about 0.35 GPa.

## 4 Discussion

High-pressure application to the Fe^3+^-doped LiGaO_2_ caused several effects on the Fe^3+^ luminescence. It is observed a shift of the luminescence maximum toward longer wavelengths accompanied by a strong decrease in the luminescence intensity, which is finally quenched at a pressure above 14 GPa. The sample also undergoes apparent phase transitions, followed finally by amorphization. Amorphization of the nanocrystallites is reversible, although only partially since after decompression, the sample does not return to the initial crystallographic orthorhombic structure.

Previously, it was observed that for Mn^2+^ dopant, having the same d^5^ electronic structure as Fe^3+^, in several materials (jervisite NaScSi_2_O_6_,^42^ pentaborate GdZnB_5_O_10_,^41^ and Tb_3_Al_5_O_12_ garnet^48^), pressure application lead to the luminescence quenching, which was associated with pressure-induced crossing between the luminescent ^4^T_1g_ emitting level with the nonluminescent, strongly coupled to the lattice ^2^T_2g_ level. Pressure-induced decrease in the decay time of Mn^2+^ in ZnS was also observed in ZnS.^49^

In all of the abovementioned cases, the luminescence efficiency quenching was accompanied by the appropriate decrease of the luminescence decay times, which confirmed the pressure-induced increase influence of the nonradiative transitions. In the case of LiGaO_2_:Fe^3+^, a strong luminescence efficiency decrease is observed with the increase of pressure; however, only a very limited decrease of the luminescence decay times occurs with the increase of pressure.

The energy of the ^4^T_1g_ level of Fe^3+^ ions can be established as a sum of the luminescence peak energy and the Stokes shift, calculated from eq 7. However, the pressure-induced phase transitions, occurring in LiGaO_2_ do not allow to use of that formula since the mechanisms leading to the pressure-induced changes of the FWHM are not only limited to the effects associated with the configurational coordinate model but involve also a contribution related to the structural crystallographic changes, especially important above 4 GPa, where amorphization of the material takes place.

**Figure 11:**
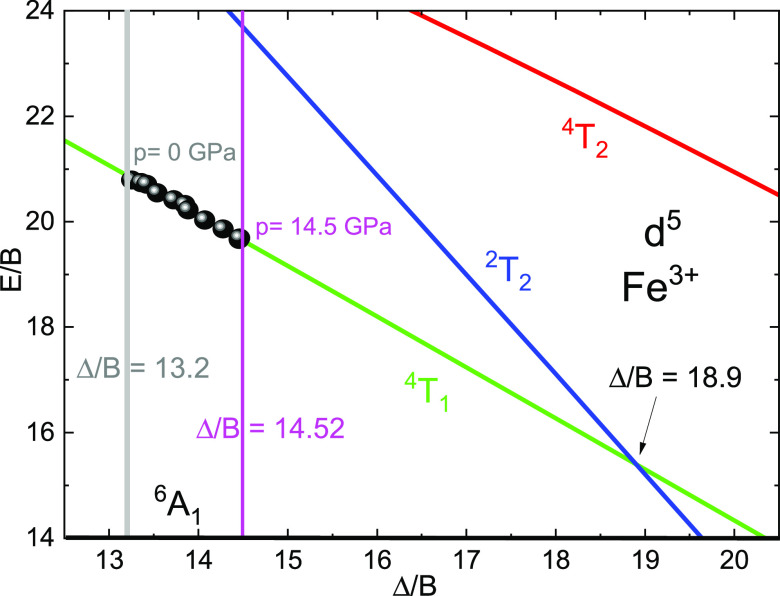
Partial Tanabe–Sugano diagram for Fe^3+^, calculated for Racah parameter *B* = 713 cm^–1^, *C*/*B* = 4.2, and 10 *Dq*/*B* = 13.1. The black points are experimental data calculated from eq 9.

On the other hand, an apparent shift of the luminescence peak energy is observed as a function of pressure (see Figures 6 and 9a). Therefore for estimation of pressure dependence of the ^4^T_1g_ level energy, we use the sum of the position of the luminescence peak, *E*_lum_(p), and the value of the Stokes shift at ambient pressure, *E*_Stokes_(0 GPa), at *T* = 7 K.
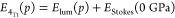
9The estimated energies of the ^4^T_1g_ level as a function of pressure were compared with the position of the ^4^T_1g_ level on the Tanabe–Sugano diagram, calculated with the proper values of the Racah and crystal-field Dq parameters. The results are shown in Figure 11.

The experimental points, calculated from eq 9 for pressures between 1.85 and 14.5 GPa span the Δ/*B* values between 13.2 and 14.52. Since the possible pressure-induced increase of the Stokes shift is not taken into account here, the span of Δ/*B* can be even smaller. As can be seen from Figure 11, the distance from the crossing point between the ^4^T_1_ and ^2^T_2_ states, which occurs at Δ/*B* = 18.9 is well separated from the position of the ^4^Τ_1_ state at the highest reached pressure *p* = 14.5 GPa. The distance between ^4^T_1_ and ^2^T_2_ states at pressures examined in our studies are also very strongly separated by almost 2800 cm^–1^ at the highest pressure (and more at lower pressures). Thus, the mixing between the nonemitting ^2^T_2g_ and emitting ^4^T_1g_ state is very small, which explains why the decay kinetics remain very weakly affected by the mixing between these states, and the decay times are very slightly disturbed by it.^48^

Nevertheless, the Fe^3+^ luminescence is quenched by the pressure applied to the host LiGaO_2_. The quenching is associated with the amorphization process occurring at higher pressures. The process of amorphization is gradual and begins at pressures around 6–7 GPa. With the increase of pressure, the amount of amorphous material increases, and since the amorphous state does not emit, finally, the emitting property of LiGaO_2_:Fe is lost.

The amorphization process is related to the loss of the layered rock salt structure of LiGaO_2_ above 3 GPa and its shifting to the disordered rock salt structure of the δ-LiGaO_2_ polymorph at high pressure.^7^ Previously measured pressure-induced quenching of the Raman was found to be related to this effect.^35^

## 5 Conclusions

The β-LiGaO_2_ sample, doped with 0.25% iron (Fe^3+^) synthesized through a high-temperature solid-state reaction method underwent thorough characterization using various spectroscopic techniques at ambient, low-temperature, and high-pressure conditions. Our investigations of the NIR luminescent β-LiGaO_2_:Fe^3+^ phosphor revealed an overall increase in its luminescence emission intensity at very low temperatures, exhibiting a millisecond lifetime and undergoing multiple irreversible phase transitions under high pressure. We observed a shift toward longer wavelengths, the far-red luminescent band associated with the ^4^T_1g_ → ^6^A_1g_ transition of the Fe^3+^ ion with an increase of pressure. At around 3 GPa pressure, the material lost its characteristic zero-phonon line (ZPL) due to a phase transition from the orthorhombic to trigonal phase. Furthermore, the primary luminescence band was completely quenched at approximately 14 GPa as a result of material amorphization. The phosphor demonstrated a quantum yield value of 28% at ambient conditions. Additionally, the LiGaO_2_:Fe^3+^ powder exhibited mechanoluminescence properties in the near-infrared spectral region, making it particularly interesting for potential application in the first biological spectral region. The pressure-induced emission changes of the LiGaO_2_:Fe^3+^ powder (between approximately 750 and 800 nm) coincided with the strong absorption changes of deoxyhemoglobin, which may be an additional point of interest for potential applications.^50^
